# Design of a Controlled-Release Delivery Composite of Antibacterial Agent Gatifloxacin by Spherical Silica Nanocarrier

**DOI:** 10.3389/fchem.2021.821040

**Published:** 2022-01-13

**Authors:** Xueping Guo, Wenjing Mo, Dingyang Zhang, Yurong Wang, Fang Cao, Tianyun Zhai, Wenhua Rao, Xiong Guan, Lei Xu, Xiaohong Pan

**Affiliations:** ^1^ State Key Laboratory of Ecological Pest Control for Fujian and Taiwan Crops and Key Lab of Biopesticide and Chemical Biology, Ministry of Education and College of Plant Protection, Fujian Agriculture and Forestry University, Fuzhou, China; ^2^ Graduate School of Chinese Academy of Agricultural Sciences, Beijing, China

**Keywords:** gatifloxacin, nano-silica, *E. coli*, antibacterial activity, microscopic investigations

## Abstract

In this study, a spherical silica nanoparticle was explored as a gatifloxacin carrier synthesized by the chemical precipitation method. It was found that there was no new chemical bond formation during the loading process between gatifloxacin and silica, which implies that the binding was driven by physical interaction. In addition, the drug loading and encapsulation efficiency could be improved by appropriately increasing nano-silica content in the loading process. Meanwhile, the release rate of gatifloxacin after loading nano-silica was also improved, suggesting the successful design of a controlled-release delivery composite. The silica nanocarrier could significantly improve the antibacterial performance of *Escherichia coli* by 2.1 times, which was higher than the pure gatifloxacin. The 24 h bacteriostatic rate was higher than that of a simple mixture of silica nanoparticles and gatifloxacin. Strong reactive oxygen species (ROS) in GAT-SiO_2_ NPs suggests that ROS might be associated with bactericidal activity. The synergy between the physicochemical effect and ROS production of this material is proposed as the mechanism of its antibacterial activity, which can also be confirmed by the cell membrane damage observed under electron microscopy and DNA damage experiments. Collectively, our finding indicates that nano-silica microspheres could serve as a promising carrier for the sustained release of gatifloxacin, thereby providing a new carrier design scheme for the improvement of the antibacterial effect.

## Introduction

Gatifloxacin (GAT) is a new type of fluoroquinolone antibiotics, which mainly inhibits DNA topoisomerase and gyrase of bacteria to achieve an antibacterial effect. It has the advantages of broad-spectrum antibacterial, low phototoxicity, and good tissue operation (Takei et al., 1998). In clinical application, it has a good antibacterial effect on Gram-negative and Gram-positive bacteria, and it is also effective on *mycoplasma* and *chlamydia* (Mitscher, 2005; Ibrahim et al., 2010). However, the bioavailability of gatifloxacin in traditional pharmaceutical dosage forms is very low when it is acted on certain and sensitive physiological environments, such as mucous membrane and cornea (Jegal et al., 2019). Consequently, increasing the dosage and frequency of drug administration may cause some side effects such as dyspnea, arrhythmia, and abnormal blood sugar (Xu et al., 2010). Therefore, developing a slowing and controlling gatifloxacin delivery system by a new drug carrier has significant research and clinical application value.

Compared with the traditional types of drug delivery carrier, nanocarrier has many advantages due to their excellent small-size effect (Davda and Labhasetwar, 2002; Stark, 2011), for example in high drug load and encapsulation rate, non-toxicity or low toxicity, obvious targeting, and long circulation time *in vivo* (Ranganathan et al., 2012; Moorthy et al., 2013). Among them, nano-silica has attracted wide attention due to its environmentally friendly and stable properties in various chemical environments. In recent years, it has been considered one of the most promising drug delivery systems and widely used in pharmaceutical preparations. Previous studies had applied the nano-silica to enhance drug release and improve the solubility of loaded drugs in solutions (Gao et al., 2011; Dement’eva et al., 2020; Qiu et al., 2021). The addition of a small amount of ultrafine silica could change the dissolution rate of griseofulvin, which could highly enhance the dispersibility of insoluble drugs in water (Rojas and Kumar, 2011). SiO_2_ nanospheres (SiO_2_ NPs) had significantly increased the loading capacity of layered copper hydroxybenzoate and prolonged the release time (Wang et al., 2019). However, to our best knowledge, there are few reports on SiO_2_ NPs as nanocarrier to delivering gatifloxacin, motivating us to explore the effect of SiO_2_ NPs on the antibacterial activity of gatifloxacin.

In the present study, we synthesized a kind of uniform spherical nano-silica by chemical precipitation and explored its possible synergistic antibacterial mechanism of gatifloxacin. The results indicate that the nano-silica could significantly improve the antibacterial effect of gatifloxacin. Nano-silica-loaded gatifloxacin can stress bacteria to produce reactive oxygen species, further mediating the DNA damage mechanism that seriously destroys the cell membrane, enhances the leakage of cytoplasmic content, and has a greater destructive effect on *Escherichia coli*. The findings of our study have provided valuable insights into the real application of nano-silica as a drug carrier.

## Materials and Methods

### Synthesis

The drug used in this experiment is the gatifloxacin capsule produced by Shandong Roxin Pharmaceutical Group Co., Ltd. Gatifloxacin powder was ground. Sodium silicate was added to sodium chloride to react and generate precipitates. Sulfuric acid was used to adjust pH in the synthesis process, and the precipitates were obtained after centrifugation. The precipitates were then placed in an oven to dry at 80°C. Prepared nano-silica sealed drying preservation.

### Characterization of SiO_2_ NPs and GAT-SiO_2_ NPs

The general morphology and size distribution of NPs were characterized by transmission electron microscope JEM-1200EX (120 kV) and scanning electron microscope (Hitachi, Tokyo, Japan). The particle size of nanoparticles was measured by dynamic light scattering (DLS), the DLS model, BI-Zeta PALS, that is, Zeta Sizer Nano S (Malvern, United Kingdom). The electric potential was used to measure the charge number of nanoparticles by dynamic light scattering. FT-IR measurements were recorded in the wave number range of 500–4,000 cm^−1^ using an FTIR-1500 Fourier Transform infrared spectrometer (Josvok, China). Sample of gatifloxacin, nano-silica, and GAT-SiO_2_ NPs and potassium bromide were mixed in a ratio of 2 : 100. The mixture was pressed mechanically into sheets and placed in an oven to dry. The dried samples were measured in the sample chamber to obtain the IR spectra. The sample should be dried during measurement. Phase analysis was performed on the synthesized nanosized silica tablets using a BRUCKER D8 (Bruker, Leipzig, Germany) X-ray powder diffractometer with a copper target at a 2*θ* angle of 10–90°. The rate is 6°, and the step length is 0.02°.

The formula is as follows:

2dsin⁡θ=nλ,

where *θ* is the incident angle, *d* is the interplanar spacing, *n* is the diffraction order, and λ is the wavelength of the incident ray.

### Drug Load and Encapsulation Rate of GAT-SiO_2_ NPs

#### Detection of Drug Loading and Encapsulation Rate of GAT-SiO_2_ NPs

To detect drug loading and encapsulation rate of GAT-SiO_2_ NPs, 0.002 g of gatifloxacin was weighed and added to four conical bottles containing 100 ml 0.1 mol/L of HCl, and 0.001, 0.0015, and 0.002 g of nano-silica were successively added. The conical flask was stirred under a magnetic stirrer at 80°C until gatifloxacin loaded with nano-silica dissolved. The content of gatifloxacin was determined by UV spectrophotometer after gatifloxacin loaded with nano-silica was fully dissolved. The detection wavelength was 291.5 nm, and the content of gatifloxacin was obtained according to the standard curve of gatifloxacin at 0.1 mol/L HCl with the nano-silica solution as the control. The formulae of drug loading and encapsulation rate are as follows (Hosny, 2010; Duxfield et al., 2016):

$${\text{S}}_{1}=\frac{m_{1}}{m_{2}}\times 100\text{\%},$$

$${\text{S}}_{2}=\frac{m_{1}}{m_{0}}\times 100\text{\%},$$

where *m*_0_ is the actual drug mass, *m*_*1*_ is the drug mass on the drug-loaded microsphere, and *m*_2_ is the mass of nano-silica carrier.

#### Releasing Ratio of GAT-SiO_2_ NPs

GAT pharmaceutical powder and GAT-SiO_2_ NPs were dissolved in 0.1 mol/L HCl respectively, placed in a shaker, and stirred evenly at 37°C. Subsequently, 10 ml of samples was taken every 30 min and centrifuged in a centrifugal tube. The absorbance of the supernatant was measured by a UV spectrophotometer, and the hydrochloric acid solution with nano-silica was used as the control. The content of gatifloxacin released into the solution was obtained by absorbance and standard curve of gatifloxacin at 0.1 mol/L HCl, from which the release rate of gatifloxacin and nano-silica loaded gatifloxacin could be obtained.

### Antibacterial Behavior

#### Effects of Different Treatments on the Growth of *E. coli*

*E. coli* was inoculated into LB liquid medium and incubated in a shaker at a constant temperature for 24 h. 10 ml of bacterial solution was taken for centrifugation, and the supernatant was decanted. Gatifloxacin, nano-silica, equal-mass mixture of gatifloxacin and silica (named GAT + SiO_2_ NPs), and GAT-SiO_2_ NPs were weighed according to M _bacterial solution_: M _drug_ = 100:1, and then the above three samples were added to the centrifuge tube containing bacterial. The other one not added to any drugs was made the control. The bacteria in the centrifuge tube were stirred for even distribution, sealed with a sealing film incubated at 37°C for 24 h. During the cultivation process, the bacterial solution was placed in a UV spectrophotometer to determine OD_600_ at 4, 6, 12, 16, 24 h, and measurements were repeated three times. The influence of four treatments on the growth of *E. coli* can be obtained by the formula of inhibition rate:

$$\text{η}=\frac{\left({\text{A}}_{0}-{\text{A}}_{1}\right)}{{\text{A}}_{0}}\times 100\text{\%},$$

where *A*_*0*_ is the OD value of the bacteria solution without any drugs and *A1* the bacteria solution with drugs.

#### Reactive Oxygen Species-Mediated DNA Damage

The production of ROS was analyzed using the sensitive dye 2′,7′-dichlorodihydroflourescein diacetate (DCFH-DA). In the experiment, 1 ml of bacterial cells treated in the above description was collected, washed, and incubated with 10 μmol/L DCFH-DA dye at 37°C for 30 min. Before the test, the bacteria were washed with PBS to remove the residual dye. The distribution of dichlorofluorescein (DCF) was observed under a laser confocal microscope. The excitation wavelength was 488 nm, and the emission wavelength was 525 nm. Subsequently, 5 ml of bacterial cells treated in the above description was collected, and DNA was extracted. DNA cleavage was analyzed by the agarose gel electrophoresis.

#### Bacterial Viability Analysis Using Fluorescence Microscopy

A mixture of equal volumes of SYTO®9 (Life Technologies) and Propidium iodide was freshly prepared (Stiefel et al., 2015) and used to stain the bacterial cells treated as described previously. After mixing thoroughly, samples were incubated at room temperature in the dark for 15 min. 20 µl of the stained bacterial suspension was trapped between a slide and an 18 mm square coverslip. Bacteria with intact cell membranes were stained fluorescent green, whereas bacteria with damaged membranes were stained fluorescent red. The excitation/emission maxima for these dyes are about 480/500 nm for SYTO^®^9 stain and 490/635 nm for propidium iodide. The background remains virtually nonfluorescent (Robertson et al., 2019).

## Results and Discussion

### Characterization

**FIGURE 1:**
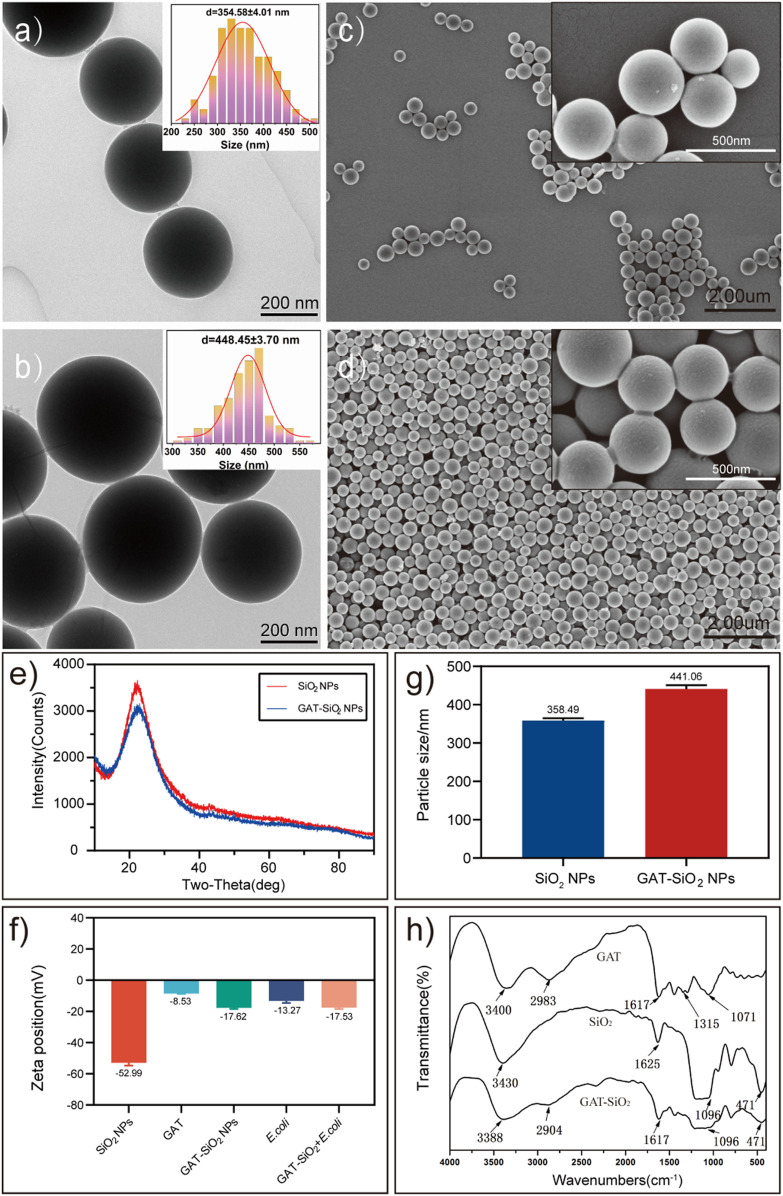
The characteristics of spherical SiO_2_ NPs and GAT-SiO_2_ NPs. **(A,B)** TEM image of SiO_2_ NPs and GAT-SiO_2_ NPs. **(C,D)** SEM image of SiO_2_ NPs and GAT-SiO_2_ NPs. **(E)** XRD pattern of SiO_2_ NPs and GAT-SiO_2_ NPs. **(F)** Zeta potential of SiO_2_ NPs and GAT-SiO_2_ NPs (mean ± SD; *n* = 3). **(G)** The average grain diameter of SiO_2_ NPs and GAT-SiO_2_ NPs (mean ± SD; *n* = 3). **(H)** The FT-IR spectra of SiO_2_ NPs, GAT, and GAT-SiO_2_ NPs.

Scanning electron microscopy and transmission electron microscopy show that the nano-silica are evenly spherical solid with a smooth surface (Figures 1A,C), and the morphology had no obvious change after loading gatifloxacin (Figures 1B,D). The X-ray powder diffraction (XRD) patterns have shown that the nano-silica with broad diffraction peaks (2*θ* = 22°) is an amorphous form (Figure 1E). However, there are no new peaks of nano-silica after loading with gatifloxacin. It was found that the average particle size increased after loading (Figure 1G), indicating that gatifloxacin was wrapped on the surface of nano-silica and formed a drug film (Pan et al., 2018). Zeta potential results (Figure 1F) have shown that the surface of the pure nano-silica solution was negatively charged (−56 mV), which is in the range providing moderate electrostatic stabilization of suspensions (−31∼ −60 mV) (Muller and Heinemann, 1993). The pure gatifloxacin solution was negatively charged (−8.53 mV). Subsequently, the solution potential of nano-silica-loaded gatifloxacin was increased to −17.62 mV, which indicated gatifloxacin stability could be improved by nano-silica, and the binding of nano-silica and gatifloxacin could not be driven by the electrostatic interactions but probably by intermolecular forces (Zhang et al., 2019). According to the characterization of SEM, TEM, particle size, and Zeta potential, we speculated that gatifloxacin could form a film on nano-silica. However, it was known that the surface of un-treatment silica is generally coated by polar groups (such as -OH) (Zhang et al., 2019), and the negative values of gatifloxacin log P indicate a high affinity of the compounds into a polar environment at a given pH (6.8). The interaction between them is based on the formation of dipole-dipole or dipole-induced dipole electrostatic interaction (Kłosińska-Szmurło et al., 2014).

Moreover, the infrared spectrum of gatifloxacin retains its characteristic peak (Figure 1H) compared with a previous study (Althubeiti, 2020). Nevertheless, the infrared spectrum of gatifloxacin shows several new peaks after binding of nano-silica. The new peaks might attribute to Si-O-Si antisymmetric stretching vibration peak at 1,096 cm^−1^ and Si-O bending vibration absorption peak at 471 cm^−1^ (Zhang et al., 2007). The -OH stretching vibration absorption peak and -CH_2_ or -CH_3_ stretching vibration absorption peak have red shift, demonstrating that no new bond was formed between gatifloxacin and nano-silica. It is speculated that the molecular interaction between gatifloxacin and nano-silica had been promoted by the formation of hydrogen bond between silica surface and N-H in gatifloxacin (Aljuffali et al., 2015). Combined with the particle size measurement results, it can further be speculated that gatifloxacin and nano-silica were bonded by physical embedding (Thewalt and Tieleman, 2016). In all, these characterizations indicated that gatifloxacin was successfully loaded on nano-silica, and the dispersibility of gatifloxacin could be improved by nano-silica.

### Drug Loading and Release Behavior

**FIGURE 2:**
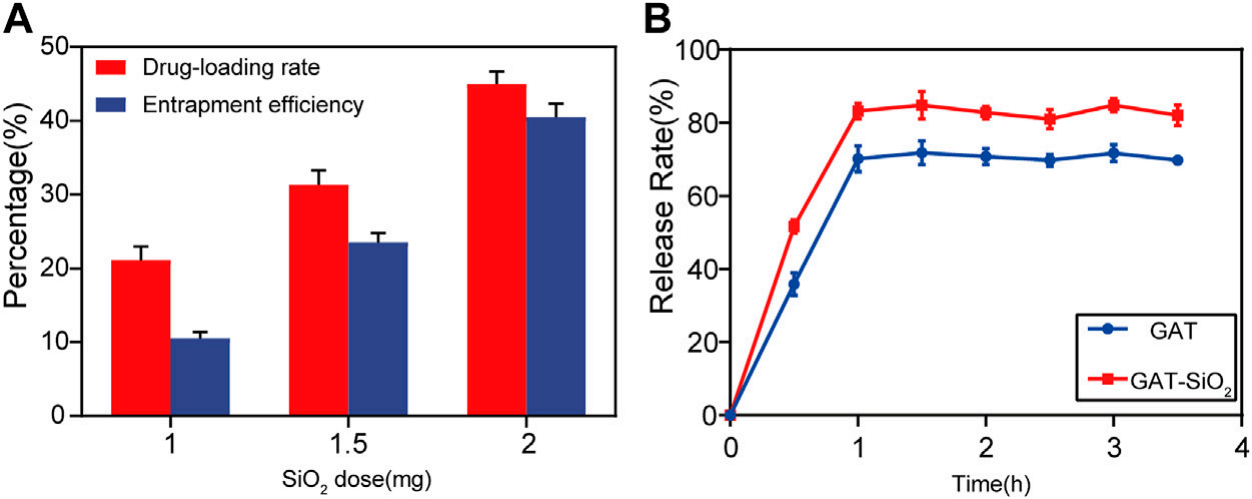
**(A)** Effects of SiO_2_ NPs dose on encapsulation efficiency and drug-loading rate of gatifloxacin (mean ± SD; *n* = 3. **(B)** The mean cumulative release (±SD) of gatifloxacin loaded by SiO_2_ NPs and pure gatifloxacin solution over a period of 4 h.

In terms of controlled-release outcomes of drugs, it was found that the drug load and encapsulation rate were greatly influenced by the dose of nano-silica (Figure 2A). As the content of nano-silica increases from 1.00 to 2.00 mg, the drug load and the encapsulation rate increase by 25 and 35%, respectively, indicating that the drug load and encapsulation rate were directly proportional to the dose of nano-silica (Mishra et al., 2017). Additionally, the release rate of gatifloxacin loaded with nano-silica was higher than that of pure gatifloxacin (Figure 2B). The release rate of both GAT and GAT-SiO_2_ NPs reached the highest after 1 h and then tended to stabilize, but the highest release rate of gatifloxacin could be increased from 72.1 to 85.3% by nano-silica loading. Moreover, the rapid initial release of gatifloxacin within 1 h may be due to the hydrophilicity of the nano-silica. The hydrophilicity of nano-silica plays an important role in this process, which makes the solution penetrate into the nanoparticles and dissolves the embedded gatifloxacin. The solubility of gatifloxacin mainly depends on pH (up to 40–60 mg/ml at pH 2.5). However, it has exhibited very low solubility at approximately physiological pH (10 mg/ml) (Motwani et al., 2008). In this sense, the loading conditions at the site of this study and the small size (large surface area) of the silica nanoparticles resulted in the initial explosive release.

### Antibacterial Behavior

**FIGURE 3:**
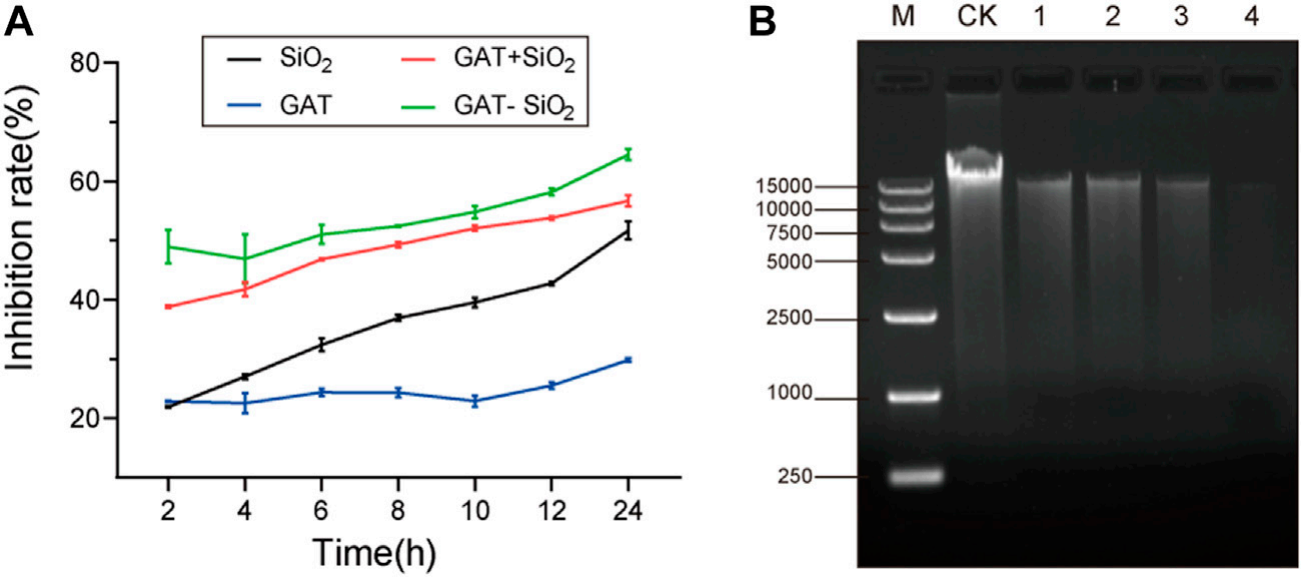
**(A)** Bacteria inhibitive rate on gatifloxacin, SiO_2_ NPs, gatifloxacin and silica simple mixture, and GAT-SiO_2_ NPs. **(B)** Agarose gel electrophoresis images of DNA extracted from different treatments of *E. coli*. CK: intact *E. coli* cells. (1): treated with SiO_2_ NPs; (2): treated with gatifloxacin; (3): treated with a mixture of gatifloxacin and silica; (4): treated with GAT-SiO_2_ NPs.

The bacteriostatic rate of *E. coli* treated with gatifloxacin, nano-silica, GAT + SiO_2_ NPs, and GAT-SiO_2_ NPs at different time intervals is shown in Figure 3A. It was found that all four treatments had a certain inhibitory effect on *E. coli*, and the inhibitory rate of *E. coli* increased gradually with the extension of treatment time. For example, the highest inhibitory rate of gatifloxacin, nano-silica, GAT + SiO_2_ NPs, and GAT-SiO_2_ NPs on *E. coli* was 29.8, 51.4, 56.7, and 64.5% at 24 h, respectively. It further suggests that the antibacterial activity of gatifloxacin could be significantly enhanced nearly three times by nano-silica and has a higher antibacterial effect than the simple mixture of the two in 24 h. Although the release of gatifloxacin is a rapid process, the antibacterial experiments indicated that GAT-SiO_2_ NPs still had a higher antibacterial rate than pure gatifloxacin at 24 h. This may be attributed to the nano-silica itself having certain antibacterial properties. Due to the large specific surface area and large number of surface hydroxyl groups, nano-silica has high reactive sites. The antibacterial activity of its preparation is mainly mediated by contact, and it has a longer-lasting antibacterial effect than a simple pharmaceutical agent (Botequim et al., 2012).

Based on the experimental results of the inhibition rate, we speculate that the reason for the synergy may be that the silica nanoparticles assisted in destroying the bacterial cell membrane (Wozniak-Budych et al., 2017). Since the disruptions of the bacterial structure and cell membrane might have caused the leakage of the intracellular contents (e.g., DNA), the effect of different treatments on the *E. coli* genome had been detected to determine whether nano-silica could increase the damage on DNA of bacteria. Hence, we evaluated the damage of bacterial DNA by agarose gel electrophoresis. A slight reduction in DNA is induced by all treatments, which is seen as a thinner band in exposed samples, compared to the thicker band of the control (DNA without treatment) (Figure 3B). The DNA band treated with GAT-SiO_2_ NPs was the thinnest, and the fragment length was reduced compared with the control, indicating that GAT-SiO_2_ NPs could significantly induce DNA damage.

**FIGURE 4:**
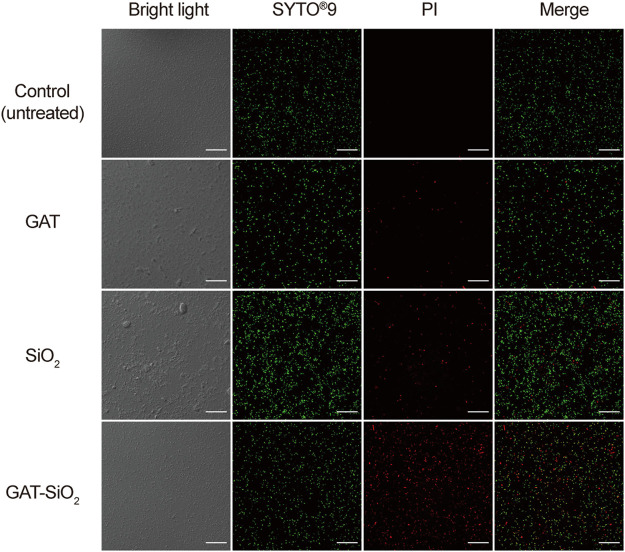
The fluorescent images are captured under CLSM after dying with DCFH-DA (green indicator). Scale bar represents 30 μm.

ROS-mediated DNA damage is considered one of the antibacterial mechanisms of most nanomaterials (Li et al., 2012). DCFH-DA is a well-known nonpolar dye converted into the polar derivative DCFH when oxidized by intracellular ROS (Rastogi et al., 2010). The fluorescence images of the control and treated samples after background subtraction are shown in Figure 4. The control sample showed almost no green color. Nano-silica and gatifloxacin exhibited week green fluorescence and produced low ROS. However, noticeably increased green fluorescence was observed in *E. coli* treated with GAT-SiO_2_ NPs and a mixture of gatifloxacin and silica. The green fluorescence of GAT-SiO_2_ NPs was significantly stronger than the mixture of the two, indicating that GAT-SiO_2_ NPs produced a significant ROS level.

**FIGURE 5:**
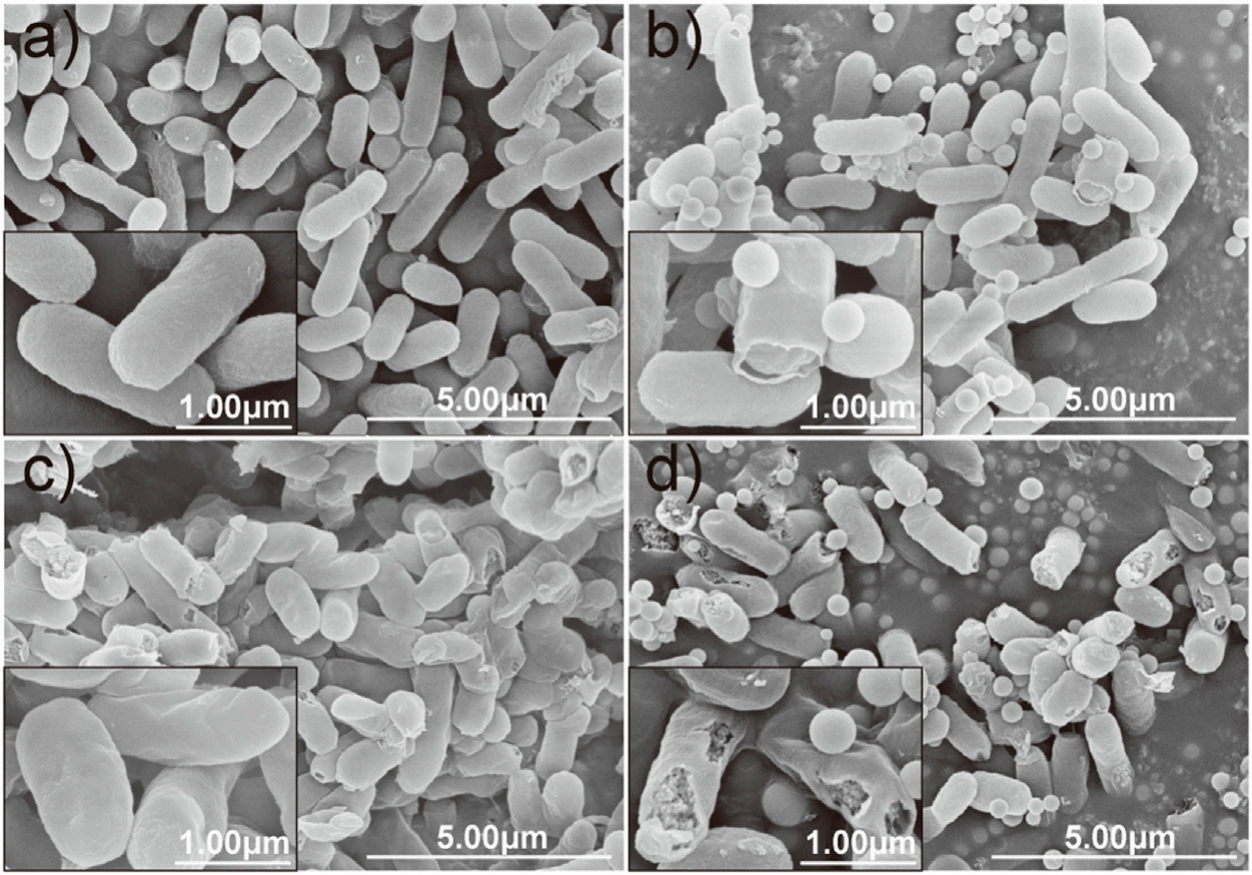
CLSM images of *E. coli* before and after treatment with SiO_2_ NPs, gatifloxacin, and GAT-SiO_2_ NPs. Live bacteria were stained with green fluorescence (SYTO^®^9). Dead bacteria were stained with red fluorescence (PI).

In order to clearly explore the differences in cell damage by different treatments, the bacteria were observed by fluorescence and electron microscope. As shown in Figure 5, the cell was stained with a Live/Dead kit. The photographed images showed that the control of *E. coli* was stained with green fluorescent, implying the cell membrane’s integrity and vitality. As for the cell treated with pure nano-silica, the cell was stained with certain red, suggesting that the nano-silica can also rupture the cell membrane and cause the death of *E. coli*. GAT-SiO_2_ NPs-treated cells showed high red fluorescence, which indicated most of the cells were dead. This result was consistent with the antibacterial behavior that GAT-SiO_2_ NPs induced the strongest toxicity to *E. coli* as mentioned above.

**FIGURE 6:**
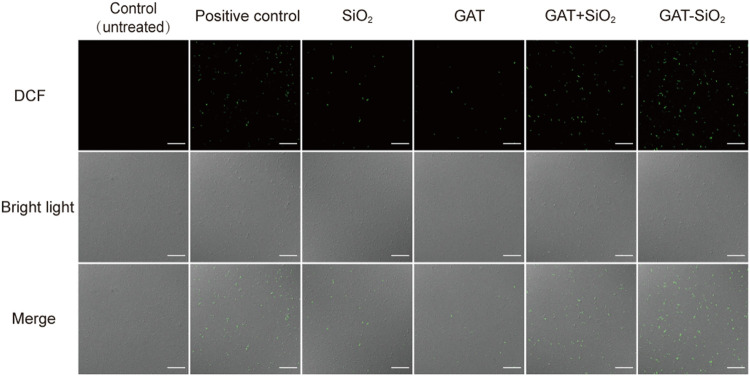
SEM images of *E. coli* in different treatment groups. **(A)** Control. **(B)** SiO_2_ NPs. **(C)** Gatifloxacin. **(D)** GAT-SiO_2_ NPs. Scale bar represents 30 μm.

Moreover, the morphological changes of *E. coli* caused by each treatment were compared and evaluated by SEM microscopy. It can be observed that the control cell of *E. coli* exhibited a relatively intact profile and clear cell walls (Figure 6A). However, the integrity of the cell membrane and cell wall of the *E. coli* was damaged by interacting with nano-silica, gatifloxacin, and GAT-SiO_2_ NPs. There are several perforations on the surface of *E. coli* after treatment with nano-silica (Figure 6B), and the bacteria were gradually crumpled and ruptured after reacting with gatifloxacin (Figure 6C). Obviously, serve damage on cell membranes and cell walls of *E. coli* was found when exposed to GAT-SiO_2_ NPs (Figure 6D), and the perforation of cells is even more serious, which leads to the leakage of contents.

**FIGURE 7:**
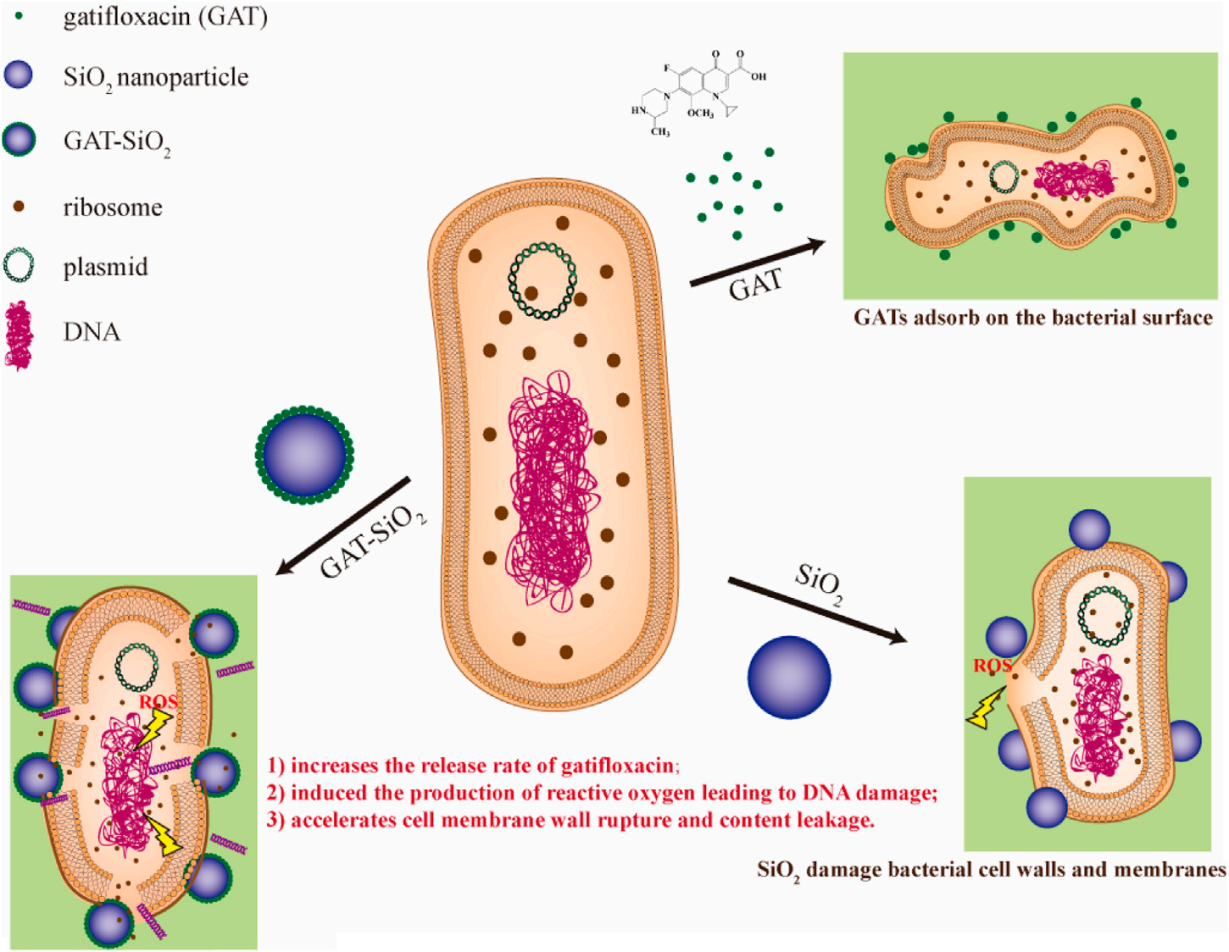
Schematic diagram of synergistic antibacterial mechanism between nano-silica and gatifloxacin.

Much evidence indicated that the antibacterial activity of nanoparticles was attributed to the disruption of bacterial membranes (Kaweeteerawat et al., 2015), production of reactive oxygen species (ROS) (Wang et al., 2017), and/or interference with the bacterial metabolism (Xiu et al., 2012). Previous studies also reported that nano-silica exhibited certain antibacterial effects (Yu et al., 2017; Priyadarsini et al., 2018). The results of this study indicate that the strong reactive oxygen species (ROS) in GAT-SiO_2_ NPs may be related to bactericidal activity. It was noted that the rich surface activity of nano-silica is in contact with the *E. coli* cell membrane to stress it to produce reactive oxygen species. This leads to oxidative stress, triggering DNA damage, destroying the cell membrane structure, and leaking out the contents. Hence, the physiological metabolism of the bacteria is inhibited or even dead. The cell membrane damage and DNA damage experiments observed under the electron microscope also support this hypothesis. Nano-silica can cause certain perforations on the surface of *E. coli*, and its antibacterial effect is better than that of gatifloxacin. However, research by Marion et al. indicated that nano-silica with the size of 100 nm diameter did not trigger any significant change in the Young modulus of *E. coli*, and the bacteria retained intact morphology and membrane structure. Combined with the observation results of SEM, bacterial inactivation occurred in the extracellular wall composed of lipopolysaccharide phospholipids and transmembrane proteins. Based on the membrane characteristics of bacteria, the maximum inhibitory efficiency of nano-silica was related to the interaction of lipopolysaccharide (Kalankesh et al., 2019). In addition, we suspect that the toxicity of the nano-silica synthesized in this study to *E. coli* depends on its charge and size (Mathelié-Guinlet et al., 2018). The subsequent modification of the mechanical properties of the bacterial surface depends on the surface properties of each object as determined by the interaction. On the contrary, silica particles do not cause cytotoxicity (Barnes et al., 2008; Yu et al., 2009). Therefore, it can be considered that the spherical nano-silica synthesized in this study has good biocompatibility with cells, with a particle size between 300 and 500 nm before and after loading. Based on the abovementioned analyses, we proposed the possible synergistic antibacterial mechanism of GAT-SiO_2_ NPs (Figure 7). First, GAT-SiO_2_ NPs with a negative charge and small size may be adsorbed to the surface of bacteria by intermolecular force. Nano-silica carrier plays an important role in improving drug stability and release rate. Furthermore, nano-silica carriers can stress bacteria to produce reactive oxygen species, resulting in oxidative stress reaction and DNA damage. Moreover, nano-silica carriers can help in increasing the damage of gatifloxacin to the cell wall, enhancing the permeability of cell membrane, leading to the outflow of cytoplasmic content, the inhibition of physiological metabolism, and even the death of *E. coli*.

## Conclusion

The nano-silica was synthesized by the chemical precipitation method with a spherical shape, smooth surface, and good physical stability. Nano-silica loaded with gatifloxacin can improve the dispersion stability of gatifloxacin in an aqueous solution and contributes to the loading and release process of gatifloxacin. When the amount of silica was 2 mg, the drug loading of gatifloxacin reached 45% and the encapsulation rate reached 40.5%. The drug loading and encapsulation rate could be increased by properly increasing nano-silica content. Moreover, the release rate of gatifloxacin was also increased after nano-silica loading, which indicates that nano-silica can help improve the utilization rate of gatifloxacin. Importantly, the antibacterial performance of nano-silica loading gatifloxacin was improved by 2.1 times. In addition, the reactive oxygen species (ROS) also plays an important role in antibacterial properties. The nano-silica could enhance the damage of cell membrane and cell wall, leading to the outflow of cytoplasmic content and DNA damage. Our finding indicates that the sustained release and synergistic antibacterial effect of gatifloxacin could be improved by nano-silica. GAT-SiO_2_ NPs can increase the release rate of the active ingredients of gatifloxacin and have a longer-lasting antibacterial effect than simple agents, which helps reduce the dosage and resistance of gatifloxacin in clinical applications.

## Data Availability

The original contributions presented in the study are included in the article/Supplementary Material. Further inquiries can be directed to the corresponding authors.
